# Effect of traditional Chinese fitness exercises on bone mineral density in postmenopausal women: a network meta-analysis of randomized controlled trials

**DOI:** 10.3389/fendo.2024.1323595

**Published:** 2024-02-06

**Authors:** Shijie Liu, Sijun Wu, Juancai Qi, Lin Wang

**Affiliations:** School of Physical Education, Wuhan University of Technology, Wuhan, China

**Keywords:** traditional Chinese fitness exercises, BMD, network meta-analysis, postmenopausal, women

## Abstract

We aimed to evaluate the clinical efficacy of five traditional Chinese fitness exercises (Baduanjin, Taijiquan, Wuqinxi, Yijinjing, and Liuzijue), as well as their efficacy when combined with drug therapy, in the treatment of decreased bone mineral density in postmenopausal women

**Methods:**

This study strictly followed the evaluation guidelines of PRISMA and followed the “PICOS” principle outlined in the Cochrane Handbook. We performed a systematic search on Web of Science, Springer Link, Scopus, EMBASE, EBSCO, PubMed, the Cochrane Library, CNKI, Wanfang, CBMdisc, and the VIP Database, and we targeted RCTs studying the effect of TCE on BMD in postmenopausal women published prior to September 2023. The quality of the literature and the risk of bias of the included studies were assessed according to ROB2 and GRADE criteria, and data analysis was performed using Stata 14.

**Results:**

A total of 33 RCTs (3658 post-menopausal women) were included. Network meta-analysis showed that Taiji (SMD=0.72, 95% CI: 0.22, 1.21, P<0.01) and Yijinjing (SMD=0.51, 95% CI: 0.03, 0.99, P<0.05) were significantly superior to conventional rehabilitation in lumbar BMD. In terms of improvement of femoral neck BMD, Baduanjin (SMD=1.63, 95% CI: -3.58, 6.85, P<0.001) and Taiji (SMD=0.46, 95% CI: 0.14, 0.79, P<0.05) had statistically different outcomes to conventional rehabilitation. Regarding Ward’s triangle BMD, Taiji (SMD= 0.32, 95% CI: 0.14, 0.50, P< 0.05) had statistically different outcomes to conventional rehabilitation. The results of the SUCRA probability ranking showed that Baduanjin + drug interventions achieved the most significant improvement in lumbar BMD (SUCRA=83.6%) and femoral neck BMD (SUCRA=90.2%). Taiji + drug interventions most effectively improved Ward’s triangle BMD (SUCRA=86.0%). In terms of traditional Chinese fitness exercises alone, Taiji was the most effective in improving lumbar BMD (SUCRA=64.4%) and Ward’s triangle BMD (SUCRA=46.8%), and Baduanjin was the most effective in treating femoral neck BMD (SUCRA=89.9%).

**Conclusion:**

Traditional Chinese fitness exercises can significantly improve the BMD levels of postmenopausal women. Taiji, Yijinjing, and Baduanjin combined with medication showed better intervention effects overall. However, due to the limitations of the number of studies and sample sizes of individual interventions, definitive conclusions need to be verified by more high-quality studies.

## Introduction

As women age, they experience degeneration of ovarian function. A lack of estrogen leads to reduced bone mass, decreased bone density, and structural changes in bone tissue, which increases bone fragility and fracture susceptibility (1–4). In an observational study, lack of estrogen increased osteoporosis-related fractures by about 50% (5). An estimated 32 million people in Europe had osteoporosis in 2019, with around 80% of cases involving postmenopausal women (6), where the residual lifetime risk of a hip fracture at the age of 50 years ranged from 7.0% (Romania) to 25.1% (Sweden) (7). As human life expectancy increases and the global aging process accelerates, the prevalence of postmenopausal osteoporosis will increase in the coming decades (8, 9). The data show that the rate of bone loss in older women is significantly accelerated 1–10 years after menopause, with an annual loss rate of 1.5–2.5% and a reduction in BMD, which increases the risk of fracture by 2.6 times (10). Recently, European data showed that menopausal women are 40% more likely to experience fractures (11). In summary, postmenopausal osteoporosis and the accompanying loss of bone density have become a severe public health problem and a threat to older women’s health.

Given the prevalence of postmenopausal osteoporosis and the many disadvantages of pharmacological treatment, such as long cycle times, high costs, adverse effects, and poor compliance, exercise therapy is gaining increasing attention as a complementary therapy to pharmacological treatment, due to its advantages of being economical and having few side effects (12). In recently years, traditional Chinese exercise (TCE), including Taiji, Yijinjing, Baduanjin, Wuqinxi, and Liuzijue, has played a significant role in the treatment of osteoporosis, and has been widely and flexibly used in clinical practice (13–15). Compared to other exercises, TCEs are easy to learn and are not restricted by exercise venues. They impact personal health and disease prevention by improving body balance (16), strengthening lower limb muscles (17, 18), and preventing postmenopausal osteoporosis (19).

Despite differences in the design of experiments testing this, TCE is a safe option for the prevention and treatment of primary osteoporosis or bone loss. Recent meta-analyses have shown that Taiji interventions prevent further osteoporotic BMD decline in elderly female patients with osteopenia or osteoporosis (20). Another meta-analysis showed that, of a range of exercises, Baduanjin was most effective in inhibiting or even reversing BMD in older adults with osteoporosis (21). However, there has been a lack of systematic reviews of TCE’s effects on BMD in older postmenopausal adults, other than Taiji. Furthermore, a recent meta-analysis suggested that different exercise patterns may affect BMD in older adults (10). Therefore, it is crucial to systematically determine the pathways of BMD influence of TCE in postmenopausal women.

Therefore, we aimed to integrate the relevant clinical evidence of the direct and indirect comparative relationships between different TCEs using a network analysis. The effect of different TCEs on BMD at different sites was assessed using a network analysis, based on a probability ranking of the superiority of the index efficacy in postmenopausal women.

## Methods

### Search strategy

This study followed the international guidelines for writing meta-analyses (PRISMA) (22). The registration number for our study is INPLASY2022110030 (DOI number 10.37766/inplasy2022.11.0030). The literature was obtained from Web of Science, Springer link, Scopus EMBASE, Cochrane Library, EBSCO, PubMed, CNKI, Wanfang, CBMdisc, and VIPdatabases. Our study aimed to identify published RCTs on the effects of TCE on BMD in postmenopausal women, with a deadline of 30 September 2023. The words used in the search of databases included “Taiji (Tai Chi or Tai Ji Quan or Taijiquan, etc.) or Health Qigong or Qigong or Qi Gong or Chi Kung or Baduanjin or Wuqinxi or Yijinjing or Liuzijue or traditional Chinese exercise AND bone density or bone mass or osteoporosis AND menopause or postmenopausal or women or female. Taking PubMed, Embase, and China Knowledge as examples, Appendix I outlines the specific search strategies.

Two researchers (S.J.L. and W.L.) independently determined the relevant research data. The degree of agreement between the two researchers was quantified using Cohen’s Kappa; the Cohen’s Kappa value of the two researchers was 0.679, indicating good agreement (23).

### Inclusion criteria and study selection

Two independent reviewers (S.J.L. and W.L.) examined the titles and abstracts of the retrieved articles and performed the primary screening based on inclusion and exclusion criteria. After obtaining the full text of the RCTs, the full text of the first selected studies was rechecked, and the studies to be analyzed were finalized. A Cohen’s Kappa value of 0.655 was obtained between the researchers, indicating moderate agreement. The studies were further reviewed by the two independent researchers until a consensus was reached. The third reviewer’s opinion (S.J.W.) was adopted without a contract.

Eligibility criteria for inclusion in the study were as follows: (1) RCTs; (2) the participants were diagnosed as postmenopausal women with osteopenia, or with normal bone mass, and had no serious complications or other diseases; (3) the experimental group included traditional Chinese fitness exercises (e.g., Taiji, Baduanjin, Wuqinxi, Yijinjing, and Liuzijue), and was compared with control groups (e.g., drugs, usual care, and exercise training); (4) outcome indicators included BMD in the lumbar spine or femoral neck, or Ward’s triangle BMD, as one of the outcome indicators; (5) before the test, each group of indicators showed a consistent baseline among subjects; (6) the study data were in the form of mean ± standard deviation or could be transformed into M ± SD (if the data in the literature were presented as the standard error (*SE*), then SD=SE× $\sqrt{N}$ (*N* is the sample size) was used for transformation); and (7) the studies were published in Chinese or English.

The exclusion criteria were: (1) duplicated studies; (2) no major TCE interventions; (3) abstract-only articles and non-RCT studies; (4) conference emails not able to be contacted; and (5) no data or data not clearly reported for analysis.

### Data extraction and quality assessment

We followed the PICO model in the reporting, design, and descriptive data extraction, including the following: first author, country, and year of publication, sample size (attrition rate), mean age or age range, years of menopause, bone mass of participant, intervention design (intervention, time, frequency), and main outcome indicators. Detailed information for inclusion in the study is shown in **Table 1**.

**Table 1:** Summary table of the studies.

Reference	Location (Language)	Participant Characteristics				Intervention Program	Intervention Characteristics			Outcome Measured	Adverse Event; Follow-Up
		Sample Size (Attrition Rate)	Mean Age or Age Range	Duration of Menopause (years)	Bone Mass of Participants		Frequency (weekly)	Time (min)	Duration (week)		
Cai. et al.(2018) (24)	Guangdong, China (Chinese)	60(0%)	EG: 51.4 ± 4.9 CG: 52.1 ± 4.2	>2	(-2.5SD<BMD≤ -1SD)	EG: Baduanjin (DT) CG: Drug group	5	60	48	①	No;No
Chen (2016) (25)	Kunming, China (Chinese)	100 (11.43%)	EG:61.2 ± 4.9 CG:60.8 ± 5.8	EG: 7.2 ± 1.5 CG: 7.5 ± 1.2	(BMD>-1SD)	EG: Baduanjin (DT) CG: Drug group	7	NR	48	① ②	No;No
Su (2018) (26)	Gansu, China (Chinese)	80(6.25%)	EG:58.93 ± 4.01 CG:59.12 ± 3.88	EG: 7.2 ± 1.5 CG: 7.5 ± 1.2	(≤2.5SD)	EG: Baduanjin (DT) CG: Drug group	5	45-60	24	①	No;No
Cheng, et al.(2017) (27),	Nanjing, China (Chinese)	65(0%)	EG: 59.4 + 6.3 CG: 58.7 + 7.9	NR	(≤2.5SD)	EG: Taiji (DT) CG: Drug group	2-3	15-20	12	① ②	No;No
Peng (2019) (28)	Gansu, China (Chinese)	72(9.72%)	EG:60.88 ± 4.59 CG:62.31 ± 4.96	EG:11.20 ± 3.16 CG:12.11 ± 3.55	(≤2.5SD)	EG: Baduanjin (DT) CG: Drug group	5	45-60	24	①	No;No
Zhou (2014) (29)	Xian, China (Chinese)	60(0%)	55.94 ± 2.83	6.58 ± 1.53	(BMD>-1SD)	EG: Taiji CG1: Exercise training CG2: Usual care	5-7	45-60	40	①	No;No
Dan (2015) (30)	Beijing, China (Chinese)	120(8.4%)	EG:60.52 ± 6.25 CG:61. 12 ± 5.87	EG:11.64 ± 5.23 CG:12.15 ± 4.67	(≤2.5SD)	EG: Taiji (DT) CG: Drug group	7	45-60	24	①	No;Yes
Liu et al. (2015) (31)	Guangzhou, China (English)	98(3.06%)	EG:61.45 ± 5.89 EG:63.23 ± 7.5 CG:62.29 ± 6.47 CG:61.87 ± 8.29	EG:11.21 ± 5.29 EG:13.79 ± 6.27 CG:12.53 ± 5.69 CG:13.24 ± 6.77	(≤2.5SD)	EG1: Baduanjin (DT) EG2: Baduanjin CG1: Drug group CG2: Usual care	3	60	48	① ②	No;Yes
Chen et al.,(2018) (32)	Jinhua, China (Chinese)	120(14%)	EG:54.80 ± 4.50 CG:55.40 ± 5.70	>1	(≤2.5SD)	EG: Wuqinxi (DT) CG: Drug group	7	20-30	48	①	No;No
Wang et al. (2018) (33)	Chaohu, China (Chinese)	86(13.4%)	EG:65.60 ± 3.80 CG:66.0 ± 4.40	10~15	NR	EG: Wuqinxi CG: Usual care	4	70	24	① ②③	No;No
Li et al. (2014) (34)	Hangzhou, China (Chinese)	60(6.67%)	EG:55.10 ± 6.52 CG:55.03 ± 5.71	EG: 8.70 ± 6.70 CG: 8.63 ± 5.60	(≤2.5SD)	EG: Wuqinxi (DT) CG: Drug group	6-7	30-60	24	①	No;Yes
Shen (2012) (35)	Guangxi, China (Chinese)	60(0%)	EG:60.44 ± 6.11 CG:60.07 ± 5.08	10.07 ± 5.39	(BMD>-1SD)	EG: Wuqinxi CG: Usual care	6	45	24	①	No;No
Gu (2021) (36)	Changsha, China (Chinese)	145(13.18%)	EG:66.36 ± 9.13 CG:65.72 ± 8.84	EG: 14.54 ± 7.54 CG: 14.87 ± 6.78	(≤2.5SD)	EG: Wuqinxi CG: Usual care	4	70	24	① ②③	No;No
Shi (2017) (37)	Hangzhou, China (Chinese)	800(18.62%)	EG:58.42 ± 4.20 CG:60.07 ± 5.08	>1	(-2.5SD<BMD≤ -1SD)	EG: Wuqinxi (DT) CG: Usual care	7	20-30	48	① ②	No;Yes
Li (2019) (38)	Chengdu, China (Chinese)	114(15.8%)	EG1:66.2 ± 3.5 EG2:65.7 ± 3.0 EG3: 65.7 ± 3.0 EG3: 65.7 ± 3.0	NR	(BMD>-1SD)	EG1: Yijingjin EG2:Wuqinxi EG3:Baduanjin CG: Usual care	5	70	48	① ②③	No;Yes
Miao (2012) (39)	Dalian, China (Chinese)	60(0%)	56.12 ± 2.96	NR	(BMD>-1SD)	EG1: Yijingjin EG2:Wuqinxi EG3:Baduanjin EG4:Liuzijue CG: Usual care	6	60	24	①	No;No
Kuo et al. (2014) (40)	Taiwan, China (English)	75(18.7%)	>50	NR	(BMD<-1SD)	EG: Taiji (DT) CG: Drug group	4-5	60	12	②	No;No
Liu, Huang (2019) (41)	Fuzhou, China (Chinese)	100(12%)	EG:55.68 ± 3.37 CG:56.92 ± 2.38	NR	正常(BMD>-1SD)	EG: Taiji CG: Usual care	3	90	16	① ②	No;Yes
Liu, Liu (2014) (42)	Xian, China (Chinese)	82(0%)	55-69	NR	正常(BMD>-1SD)	EG: Taiji CG1: Exercise training CG2: Usual care	>3	30-45	96	①	No;No
Mao et al.(2009) (43)	Xian, China (Chinese)	80(0%)	56.78 ± 2.91	6.78 ± 3.04	骨量减少(-2.5SD<BMD≤ -1SD)	EG1: Taiji (DT) EG2: Taiji CG1: Drug group CG2: Usual care	7	30	20	①	No;No
Song et al.(2018) (44)	Dazhou, China (Chinese)	146(17.8%)	EG:64. 3 ± 3. 2 CG:64. 7 ± 4. 1 CG:64. 8 ± 2. 9	NR	正常(BMD>-1SD)	EG: Taiji CG: Exercise training CG: Usual care	5	70	48	① ②③	No;Yes
Xu et al.(2017) (45)	Zhejiang, China (Chinese)	86(0%)	EG:56.2 ± 5.6 CG:57.1 ± 6.0	EG: 7.4 ± 2.7 CG: 7.6 ± 2.9	(BMD>-1SD)	EG: Taiji CG: Usual care	6	40	48	① ②③	No;No
Ye et al.(2016) (46)	Beijing, China (Chinese)	50(15%)	EG:55.38 ± 6.08 CG:57.02 ± 8.47	>2	(BMD>-1SD)	EG: Taiji CG: Usual care	3	60	24	① ②③	No;No
Yu et al.(2014) (47)	Shanghai, China (Chinese)	117(0%)	EG:55.38 ± 6.08 CG:57.02 ± 8.47	NR	(-2.5SD<BMD≤ -1SD)	EG: Taiji CG: Usual care	4	60	48	① ②③	No;No
Zhao,Cheng (2020) (48)	Chengdu, China (Chinese)	250(0%)	EG:66. 0 ± 2. 6 CG:65. 8 ± 5. 0 CG:65. 7 ± 3. 2	NR	(-2.5SD<BMD≤ -1SD)	EG: Taiji CG1: Exercise training CG2: Usual care	5-7	50-60	12	① ②③	No;No
Zhao et al. (2015) (49)	Xian, China (Chinese)	60(0%)	EG:58.8 ± 3.2	EG: 8.83 ± 0.78 CG: 8.91 ± 0.81	NR	EG: Taiji CG: Usual care	6	55	24	① ②③	No;No
Zhou et al. (2015) (50)	Xian, China (Chinese)	64(0%)	57.21 ± 3.41	6.58 ± 1.53	(-2.5SD<BMD≤ -1SD)	EG1: Taiji (DT) EG2: Taiji CG1: Drug group CG2: Usual care	5	45-60	24	①	No;No
Chan et al.,(2004) (51)	Hongkong, China (English)	132(16.9%)	EG:54.4 ± 3.3 CG:53.6 ± 3.2	EG: 4.9 ± 2.5 CG: 4.5 ± 2.4	(BMD>-1SD)	EG: Taiji CG: Usual care	5	50	48	①	No;Yes
Wayne et al.,(2012) (52)	Boston, USA (English)	86(0%)	EG:58.8 ± 5.6 CG:53.6 ± 3.2	>1	(≤2.5SD)	EG: Taiji (DT) CG: Drug group	3-5	60	36	① ② ③	No;Yes
Wang et al.(2015) (53)	Shanghai, China (Chinese)	79(0%)	EG:58.54 ± 3.37 CG:58.54 ± 3.37	>0.5	(-2.5SD<BMD≤ -1SD)	EG: Taiji CG: Usual care	4	60	48	① ② ③	No;Yes
Liu, Liu (2021) (54)	Wuhan, China (Chinese)	52(1.9%)	56.48 ± 3.41	5~8	(BMD>-1SD)	EG: Taiji CG: Usual care	3	60	48	① ② ③	No;No
Li et al.(2022) (55)	Gdan´sk, Poland (English)	32(6.5%)	EG: 57.31 ± 1.48 CG: 56.41 ± 1.68	EG: 6.23 ± 2.21 CG: 6.44 ± 1.81	(≤-2.5SD)	EG: Baduanjin (DT) CG: Drug group	5	45	16	① ②	No;No
Cheng (2020) (55)	Chengdu, China (English)	52(23.5%)	EG: 61.5 ± 3.0 CG: 61.9 ± 2.5	NR	(BMD>-2.5SD)	EG: Taiji CG: Usual care	5	60	48	① ② ③	No;No

NR, not reported; EG, experimental group; CG, control group; osteopenia = (-2.5SD<BMD≤ -1SD; osteoporosis = (≤-2.5SD); normal = (BMD>-1SD); ① lumbar bone mineral density; ② femoral neck bone mineral density; ③ Ward’s triangle bone density.

This study assessed the methodological quality of the randomized controlled trials included in the survey using RoB2, which considered the following domains: bias during randomization, inclination to deviate from established interventions (including the effect of intervention allocation and the impact of intervention adherence), bias related to missing outcome data, bias related to measurement of outcomes, and prejudice related to reporting the results selectively. Under each domain, there are multiple “signaling questions”, each of which provides five answers: Yes, Probably Yes, Probably No, No, and No Information, and based on the answers to the signaling questions, RoB2 generates a recommended risk assessment outcome.

Two independent reviewers (S.J.L. and W.L.) performed a quality assessment of the literature, and based on the RoB2 risk assessment, the included randomized controlled trials were classified as “high risk of bias”, “some concerns”, and “low risk of bias”.

In addition, we invited the first two independent reviewers to review the literature in which there was inconsistency in opinion until a consensus was reached. In the absence of agreement, the third reviewer’s opinion (S.J.W.) was adopted.

### Statistical analysis

A traditional meta-analysis was performed by applying the MetaXL program (version 5.3). Given the small sample size of the screened and suitable literature and the slight differences in evaluation tools and units used in individual studies, the standardized mean difference (SMD) and its 95% confidence interval (CI) calculated by the Hedges’g method were used to estimate the relative effect of each intervention. For each direct comparison, effect sizes were aggregated using an inverse heterogeneity (IVhet) model, which corrects for heterogeneity and generates more robust results relative to traditional fixed- and random-effects models.

Both the Q-test and *I*^2^ results determined heterogeneity in the literature. The meta-analysis was performed using a fixed-effects model when *P*≥0.1 for the Q-test and *I*^2^<50% indicated that inter-study heterogeneity was within acceptable limits, and vice versa using the random-effects model. The outcomes were categorized as either very low (<25%), low 25–50%), moderate (50–75%), or significant (>75%) (56). Furthermore, publication bias was measured using Doi plots with the LFK index, which has been shown to have higher accuracy than traditional funnel plots with the egger test, especially when the number of studies is small. Finally, sources of heterogeneity were sought through sensitivity analyses (rejected one by one) and subgroup analyses.

A network meta-analysis was performed using Stata 14.0 in a frequency-based framework. A grid relationship diagram was drawn by network meta-analysis (in the diagram, each node indicates the intervention, the size of the area of the node indicates the number of samples corresponding to the intervention, and the thickness of the line connecting the nodes indicates the number of studies included for the intervention) (57). When there was a closed-loop structure between interventions, inconsistency tests were required, and the model type was selected accordingly. If the lower limit of the 95% CI of the inconsistency factor’s (IF) value was zero (or close to zero), this indicated that the direct evidence and indirect evidence were consistent (58). Finally, the area under the cumulative ranked probability curve (surface under the cumulative, SUCRA) showed the magnitude of the likelihood of each intervention being the best intervention (56).

### Evidence certainty assessment

The Grading Recommendations to Assess Development and Evaluation system (GRADE) is an evidence evaluation system, and is one of the international standards for evidence quality and the classification of recommendation strength (13). We evaluated the quality of the evidence for each outcome using the GRADE classification with four possible levels: I (high), where the real effect is similar to a credible estimate; II (moderate), where the true effect is closest to the estimated effect; III (low), where the actual effect may be significantly different from the estimated effect; and IV (very low), where the actual effect is likely to be significantly different from the estimated effect. Five factors can cause the quality of the evidence to decrease: (1) risk of bias; (2) imprecision; (3) inconsistency; (4) indirectness; and (5) publication bias (59).

## Results

### Literature search

**Figure 1:**
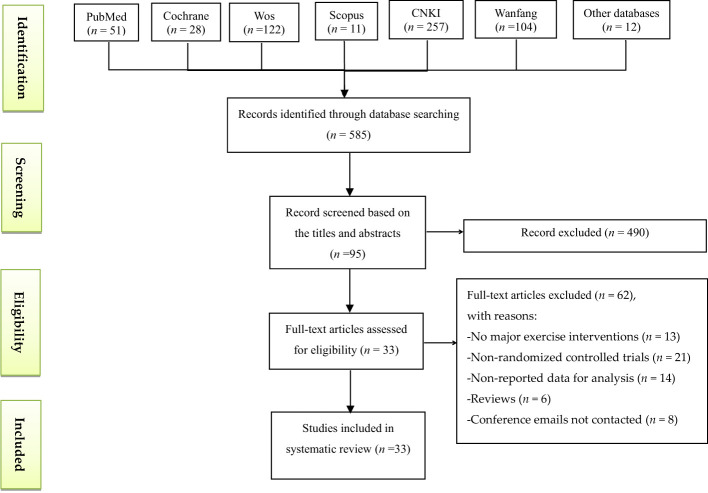
Flow of study selection.

A total of 585 articles were found from 11 databases (**Figure 1**), and 490 were removed by title and abstract. The remaining 95 articles were further screened by reading the complete text, and 62 records were excluded as they were non-RCTs (n=21), they had no reported data for analysis (*n* = 14), they were reviews (*n* = 6), they had no major TCE interventions (*n* = 13), and conference emails were not provided (*n* = 8). Finally, 33 studies were included in our network analysis.

### Study characteristics

In total, there were 3658 participants [the smallest sample was 30 (60) and the largest sample was 800 (37)]. The ages of the participants ranged from 50 to 70 years old. Three studies had an intervention period of 12 weeks (27, 40, 48), and 13 studies had an intervention period of 48 weeks, in addition to one study with an intervention period of 96 weeks (42). The experimental group involved 62 cases of traditional Chinese fitness exercises in a single treatment protocol, of which 15, 3, 5, 2, and 1 group used Taiji, Baduanjin, Wuqinxi, Yijinjing, and Liuzijue techniques as interventions, respectively. The protocols using Taiji, Baduanjin, and Wuqinxi in combination with medication included 5, 6, and 3 groups, respectively. The control groups were treated with usual care or drug therapy. Furthermore, 32 articles metrics measured lumbar spine BMD, 20 articles measured femoral neck BMD, and 13 articles measured Ward’s triangle BMD. During the intervention period, 11 articles (30, 31, 34, 37, 38, 41, 44, 51–53, 61) reported the follow-up status.

### Study quality assessment

**Figure 2:**
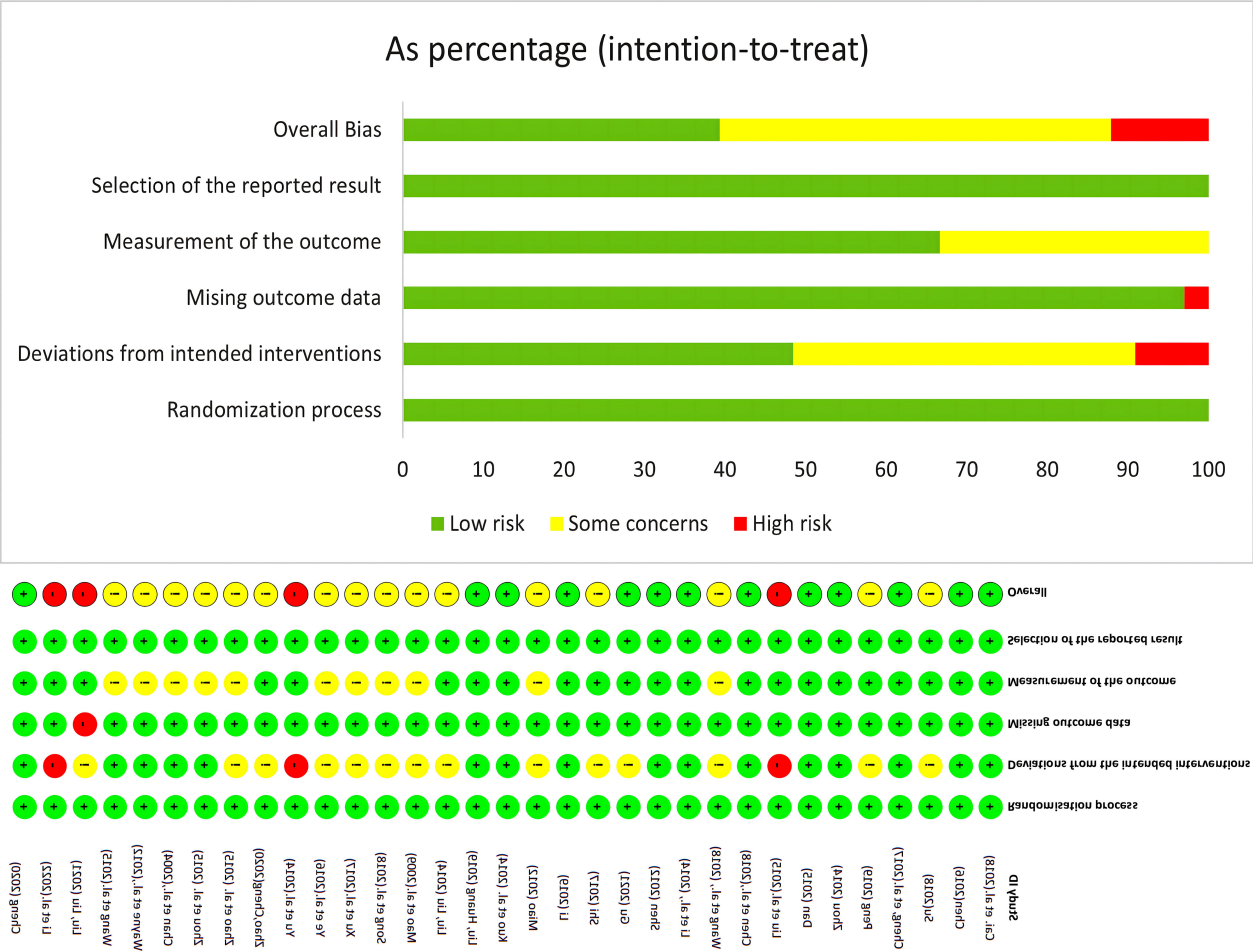
Graph of Cochrane Risk Bias Assessment.

The results of the risk of bias evaluation of the RCTs are shown in **Figure 2**. Thirty-three articles in our study reported the random allocation method as a random number table method/computer randomized generation, sixteen articles reported deviation from the intended intervention, one paper was missing outcome data, and no selective outcomes were reported in any article.

### GRADE quality evaluation

Based on the criteria of GRADE, the assessment of the certainty of the evidence regarding the significant impact of traditional Chinese fitness exercises on bone mineral density in postmenopausal women was carried out separately (**Table 2**). Specifically, traditional Chinese fitness exercises has medium-high quality evidence for the lumbar spine, femoral neck, and Ward’s triangle BMD of postmenopausal women, indicating that the quality assessment of this study had a high degree of credibility.

**Table 2:** Grading Recommendations to Assess Development and Evaluation system (GRADE) assessment of the evidence of certainty for traditional Chinese fitness exercises.

Outcomes	Presence of downgrading item of GRADE					Level of certainty of evidence
	Risk of bias	Inconsistency	Indirectness	Imprecision	Publication bias	
lumbar spine BMD	No	No	No	Yes	No	II (Moderate) (4)
femoral neck BMD	No	No	No	No	No	I (High)
Ward’s triangle BMD	No	No	No	Yes	No	II (Moderate) (4)

(1) Risk of bias: if the risk of bias of the included studies is present in the meta-analysis, e.g., randomization, concealed allocation, or blinding of assessors/subjects; (2) Inconsistency: point estimates are concentrated, confidence intervals can overlap, and the results of the heterogeneity tests are not statistically significant; (3) Indirectness: present if the intervention studied in the meta-analysis is not directly relevant to the outcome; (4) Imprecision: present if the sum of sample sizes of all individual studies included in meta-analysis is less than 500, and if the effect size’s 95% Cl is comparatively large; (5) Publication bias: present if the author only searched the Chinese database, or only one database.

### Traditional meta-analysis

#### Heterogeneity test

A total of 33 articles were included in the traditional meta-analysis, which used Doi plots and the LFK index. The LFK index in the ±1 interval suggests that there may have been minimal publication bias, in the ±2 interval suggests that there may have been a slight publication bias, and outside the ±2 interval suggests that there may have been a significant publication bias (62). Specifically, as shown in **Table 3**, in the Lumbar Spine BMD and Femoral Neck BMD indexes, the publication bias of the interventions involving Baduanjin was more significant, and in general, the studies on the effects of Baduanjin on each index of BMD in postmenopausal women had a significant bias. In contrast, the bias of studies of other Chinese traditional fitness exercises was negligible. Further heterogeneity analysis revealed that the heterogeneity of Lumbar Spine BMD and Femoral Neck BMD was significant *(I*^2^ > 50%, *P*< 0.1), and the heterogeneity of Ward’s triangle BMD was weak (*I*^2^< 50%, *P* > 0.1). Based on the data in this study, subgroup analyses were conducted by classifying different exercise forms, and sensitivity analyses (article-by-article elimination) were performed to find the source of heterogeneity (**Table 4**), which showed that except for the subgroups of Taiji and Baduanjin, which were still heterogeneous, the rest of the groups were homogeneous. A random effects model was used for the analysis to ensure the study’s accuracy.

**Table 3:** LFK index.

Indicator	Subgroup	LFK index
Lumbar Spine BMD	BDJ(DG)-Drug Group	3.16
	WQX(DG)-Drug Group	1.24
	TC(DG)-Drug Group	0.62
	BDJ-Usual Care	2.28
	WQX-Usual Care	1.41
	TC-Usual Care	1.44
	YJJ-Usual Care	NR
	LZJ-Usual Care	NR
	Exercise Training-Usual Care	-1.47
	Drug Group-Usual Care	1.74
Femoral Neck BMD	TC(DG)-Drug Group	0.56
	BDJ(DG)-Drug Group	5.42
	WQX(DG)-Drug Group	NR
	TC-Usual Care	-0.68
	BDJ-Usual Care	NR
	WQX-Usual Care	1.80
Ward’s Triangle BMD	TC-Usual Care	-0.59
	WQX-Usual Care	0.56
	YJJ-Usual Care	NR
	BDJ-Usual Care	NR

NR, not reported.

**Table 4:** Heterogeneity test.

Indicator	Subgroup	Culling before		After culling	
		*I*^2^/%	*P*	*I*^2^/%	P
Lumbar Spine BMD	BDJ(DG)-Drug Group	96.63	0.000	96.63	0.000
	WQX(DG)-Drug Group	37.78	0.201	0	0.670
	TC(DG)-Drug Group	0	0.691	0	0.691
	Summary	94.10	0.000	94.10	0.000
	BDJ-Usual Care	98.53	0.000	77.91	0.033
	WQX-Usual Care	0	0.857	0	0.857
	TC-Usual Care	90.04	0.000	77.63	0.000
	YJJ-Usual Care	0	0.970	0	0.970
	LZJ-Usual Care	NR	NR	NR	NR
	Exercise Training-Usual Care	17.49	0.303	0	0.433
	Drug Group-Usual Care	96.75	0.000	97.98	0.000
	Summary	90	0.000	86.48	0.000
Femoral Neck BMD	TC(DG)-Drug Group	51.74	0.125	0	0.636
	BDJ(DG)-Drug Group	98.09	0.000	94.08	0.000
	WQX(DG)-Drug Group	NR	NR	NR	NR
	Exercise Training-Drug Group	NR	NR	NR	NR
	Summary	91.50	0.000	53.15	0.014
	TC-Usual Care	68.59	0.001	64.63	0.001
	BDJ-Usual Care	98.86	0.000	97.51	0.000
	WQX-Usual Care	0	0.960	0	0.960
	Exercise Training-Usual Care	0	0.986	0	0.986
	YJJ-Usual Care	NR	NR	NR	NR
	Drug Group-Usual Care	NR	NR	NR	NR
	Summary	90.30	0.000	86.12	0.000
Ward’s Triangle BMD	TC-Usual Care	29.26	0.175	4.85	0.394
	WQX-Usual Care	0	0.756	0	0.756
	YJJ-Usual Care	NR	NR	NR	NR
	BDJ-Usual Care	NR	NR	NR	NR
	Exercise Training-Usual Care	0	0.372	0	0.372
	Summary	5.40	0.391	5.40	0.391

NR, not reported.

#### Traditional meta-analysis results

Traditional Chinese fitness exercises significantly improved L-BMD (SMD = 0.58, 95% CI -0.34 to 0.82, *p*< 0.01) and Femoral Neck BMD (SMD = 0.63, 95% CI 0.28 to 0.98, *p*< 0.01) in postmenopausal women, with some statistically significant improvement in Ward’s triangle BMD (SMD=0.26, 95% CI 0.15 to 0.36, *p*< 0.05) (**Table 5**). Subgroup analyses based on different exercise modalities showed that Taiji had a high effect size for improvement in L-BMD (SMD=0.72, 95% CI 0.22 to 1.21, *p*< 0.01) and a medium effect size for improvement in Femoral Neck BMD (SMD=0.46, 95% CI 0.14 to 0.79, *p*< 0.01) and Ward’s triangle BMD (SMD = 0.32, 95% CI 0.14 to 0.50, *p*< 0.01). Baduanjin had a high effect size for the improvement effect on Femoral Neck BMD (SMD = 1.63, 95% CI -3.58 to 6.85, p< 0.01) in postmenopausal women, and small effect sizes for both Ward’s triangle BMD (SMD = 0.19, 95% CI - 0.39 to 0.78, *p*< 0.05), and L-BMD (SMD = 0.03, 95% CI - 1.05 to 1.10, *p* > 0.05), which were not statistically different. Wuqinxi had a slight effect size for improvement on all BMD indicators in postmenopausal women (*p<* 0.05). Yijinjing had a significant improvement effect in L-BMD (SMD = 0.51, 95% CI 0.03 to 0.99, *p*< 0.01) and a minor improvement effect in Femoral Neck BMD (SMD=0.23, 95% CI 0.35 to 0.80, *p*< 0.05).

**Table 5:** Results of Traditional Meta-Analysis.

Indicator	Subgroup	Amount	SM D(95%CO	Forest Plot	Q	*P*	*I-suqare*
Lumbar Spine BMD femoral neck BMD Ward's triangle BMD	BDJ(DG)-Drug group WQX(DG)-Drug gro up TC(DG)-Drug group Summary BDJ-Usual Care WQX-Usual Care TC-Usual Care YJJ-Usual Care LZJ-Usual Care Summary TC(DG)-Drug group BDJ(DG)-Drug gro up WQX(DG)-Drug group Summary TC-Usual Care BDJ-Usual Care WQX-Usual Care YJJ-Usual Care Summary TC-Usual Care WQX-Usual Care YJJ-Usual Care BDJ-Usual Care Summary	5 3 5 13 3 5 15 2 1 26 3 3 1 7 9 2 3 1 15 10 3 1 1 5	1.73(0.20,3.26) 0.24(-0.02,0.49 0.22(0.00,0.43) 1.05(0.54,1.55) 0.03(-1.05,1.10) 0.18(-0.04,0.4) 0.72(0.22,1.21) 0.51(0.03,0.99) 0.44(-0.39,1.27) 0.58(0.34,0.82) 0.09(-0.34,0.51) 1.1(-2.66,4.86) 0.05(-0.09,0.19 1.09(0.30,1.88) 0.46(0.14,0.79) 1.63(-3.58,6.85 0.18(-0.07,0.43) 0.23(-0.35,0.8) 0.63(0.28,0.98) 0.32(0.14,0.50) 0.12(-0.13,0.38) 0(-0.57,0.57) 0.19(-0.39,0.78) 0.26(0.15,0.36)	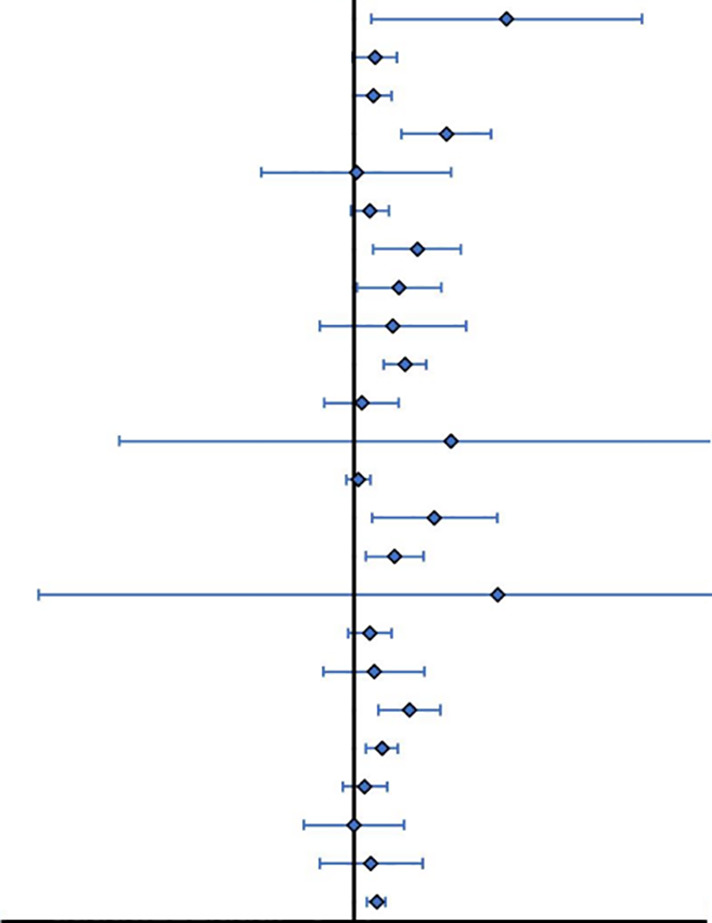	81.33 3.22 2.24 120.4 4.528 1.322 58.12 0.00 / 246.35 4.14 104.74 / 142.83 5.47 88.25 0.08 / 174.56 12.72 0.56 / / 17.97	0.000 0.200 0.692 0.000 0.033 0.858 0.000 0.970 / 0.000 0.126 0.000 / 0.000 0.103 0.000 0.960 / 0.000 0.176 0.757 / / 0.39	96.63 37.78 0.00 94.10 77.91 0.00 90.04 0.00 / 89.9 51.74 98.09 / 95.8 68.59 98.86 0.00 / 90.30 29.26 0.00 / / 5.40

Meaning of the symbol “/” stands for NR (not reported).

### Network meta-analysis

#### Effect of TCE on L-BMD in postmenopausal women

The overall inconsistency test showed that the L-BMD outcome indicator *P* > 0.05, which indicated that the overall consistency was good. Further tests of the consistency of each closed loop showed that the value of the inconsistency factor (IF) ranged from 0.06 to 0.14, and the lower limit of the 95% CI all contained 0, which indicated that the consistency of each closed loop was better, so the consistency model was used for analysis.

**Figure 3:**
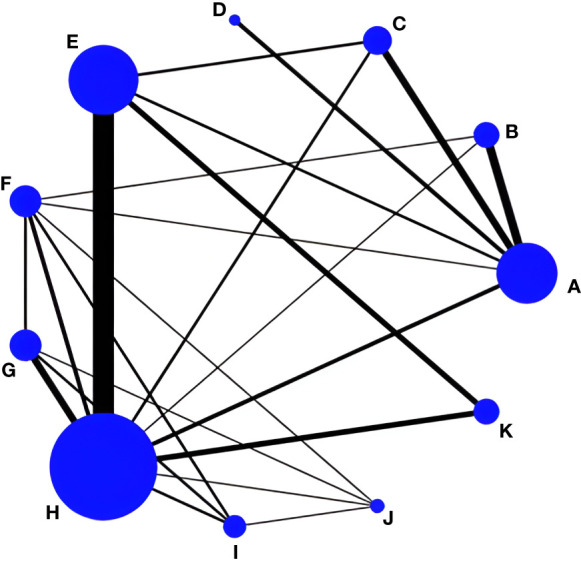
Network evidence for L-BMD. **(A)** Drug group; **(B)** Baduanjin+Drug group; **(C)** Taiji+Drug group; **(D)** Wuqinxi+Drug group; **(E)** Taiji; **(F)** Baduanjin; **(G)** Wuqinxi; **(H)** Usual care; **(I)** Yujinjin; **(J)** Liuzijue; **(K)** Exercise training.

In total, 32 RCT studies reported changes in L-BMD pretest-posttest intervention in 2771 subjects; 14 studies included drug therapy as the control group and 18 studies had usual care as the control group, with three studies reporting both drug therapy and usual care, incorporating a web of relationships between studies (**Figure 3**). The pairwise comparison between the two found that Taiji was superior to exercise training (SMD=-0.05, 95% CI -0.10 to -0.02, *p*<0.05) and Wuqinxi (SMD=-0.05, 95% CI -0.13 to -0.01, *p*<0.05) and Baduanjin (SMD=-0.06, 95% CI -0.16 to -0.02, *p*<0.05). It is worth noting that the Baduanjin+drug group was better than Baduanjin (SMD=-0.11, 95% CI -0.24 to -0.04, *p*<0.05), and the drug group (SMD=-0.08, 95% CI -0.14 to -0.02, *p*<0.05) (**Table 6**).

**Table 6:** Results of the network meta-analysis for L-BMD.

	Drug group	Exercise training	Liuzijue	Yijinjin	Usual care	Wuqinxi	Baduanjin	Taiji	Wuqinxi+ drug group	Taiji+drug group
Drug group										
Exercise training	-0.02 (-0.12,0.08)									
Liuzijue	-0.01 (-0.18,0.15)	0 (-0.17,0.17)								
Yijinjin	-0.04 (-0.16,0.09)	-0.02 (-0.15,0.11)	-0.02 (-0.19,0.14)							
Usual care	0.06 (-0.01,0.13)	0.08 (0.00,0.15) *	0.07 (-0.08,0.23)	0.20 (0.01,0.51) *						
Wuqinxi	0.01 (-0.09,0.11)	0.03 (-0.08,0.13)	0.02 (-0.13,0.18)	0.04 (-0.07,0.16)	-0.05 (-0.13,0.03)					
Baduanjin	0.02 (-0.10,0.13)	0.05 (-0.06,0.16)	0.03 (-0.18,0.23)	0.04 (-0.04,0.08)	-0.03 (-0.11, 0.03)	0.08 (-0.08,0.12)				
Taiji	-0.04 (-0.24,0.03)	-0.05 (-0.10, -0.02) *	-0.09 (-0.21, -0.04) *	0 (-0.12,0.11)	-0.29 (-0.44, -0.06) *	-0.05 (-0.13, -0.01) *	-0.06 (-0.16,-0.02) *			
Wuqinxi+ drug group	-0.02 (-0.11,0.07)	0 (-0.14,0.13)	-0.01 (-0.20,0.18)	0.02 (-0.14,0.17)	-0.08 (-0.20,0.04)	-0.03 (-0.17,0.11)	-0.05 (-0.19,0.08)	0.02 (-0.10,0.14)		
Taiji+ drug group	-0.02 (-0.08,0.05)	0 (-0.10,0.11)	0 (-0.17,0.17)	0.02 (-0.11,0.15)	-0.07 (-0.15, -0.02) *	-0.02 (-0.13,0.08)	-0.05 (-0.15,0.06)	0.02 (-0.06,0.11)	0 (-0.11,0.12)	
Baduanjin+ drug group	-0.08 (-0.14, -0.02) *	-0.06 (-0.17,0.05)	-0.06 (-0.23,0.11)	-0.04 (-0.17,0.09)	-0.14 (-0.22, -0.05) *	-0.03 (-0.19,0.03)	-0.11 (-0.24,-0.04) *	-0.01 (-0.13,0.02)	-0.06 (-0.17,0.05)	-0.06 (-0.15,0.03)

shows that the data differ. *p< 0.05.

**Figure 4:**
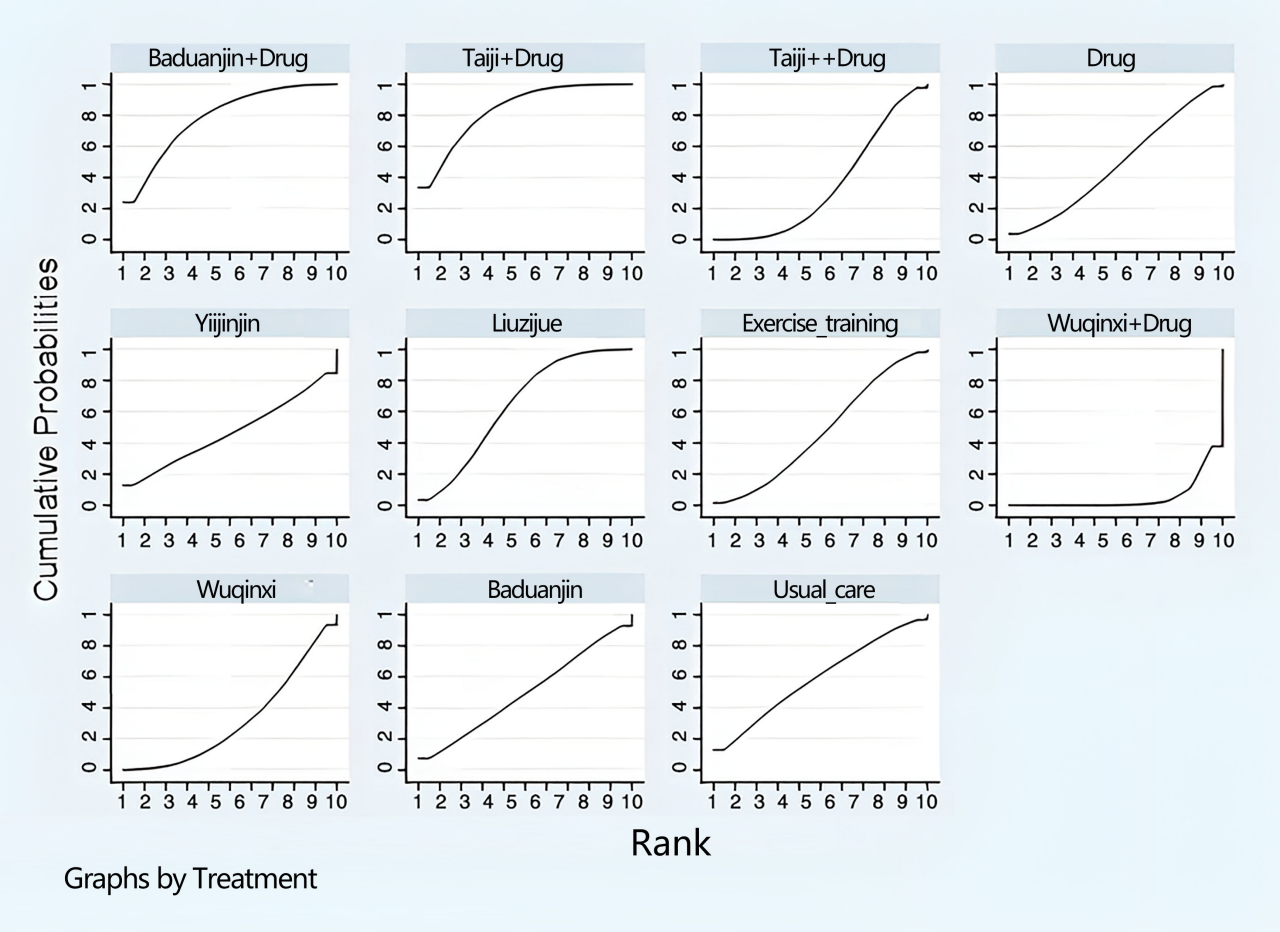
Ranking diagram of each intervention (L-BMD).

In addition, the final ranking of the five single modulation interventions, the three-drug combination interventions, and the three control groups was performed using SUCRA, with percentages indicating that the more significant the portion of the area under the SUCRA curve, the better the treatment effect (**Figure 4**). The final ranking was as follows: Baduanjin+drug (83.6%) > Taiji+drug (78.7%) > Taiji (64.4%) > drug (59.3%) > Yijinjin (49.7%) > Liuzijue (48.2%) > exercise training (47.1%) > Wuqinxi+drug (46.9%) > Wuqinxi (35%) > Baduanjin (32%) > usual care (5.2%).

#### Effect of TCE on femoral neck BMD in postmenopausal women

**Figure 5:**
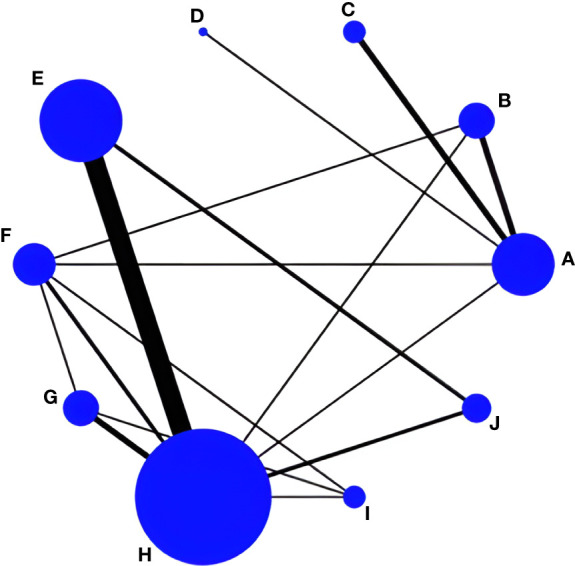
Network evidence for femoral neck BMD. **(A)** Drug group; **(B)** Baduanjin+Drug group; **(C)** Taiji+Drug group; **(D)** Wuqinxi+Drug group; **(E)** Taiji; **(F)** Baduanjin; **(G)** Wuqinxi; **(H)** Usual care; **(I)** Yujinjin; **(J)** Exercise training.

Twenty RCT studies reported changes in femoral neck BMD before and after the intervention, amounting to 2593 subjects; six included drug therapy as the control group and 13 included usual care, of which one study reported both drug therapy and usual care, and the reticulation between the included studies was performed as in **Figure 5**. Subsequently, the overall inconsistency test showed the femoral neck BMD outcome indicator *P*<0.05 and the overall consistency was poor, so the inconsistency model was used for analysis.

An indirect comparison between the two found that Taiji was superior to Yinjin (SMD= -0.07, 95% CI -0.12 to -0.05, *p*<0.05) and Wuqinxi (SMD= -0.07, 95% CI -0.13 to -0.04, *p*<0.05), but compared with Baduanjin and Baduanjin+drug, the Taiji+drug intervention effect was slightly worse. In addition, Baduanjin was superior to Taiji (SMD= 0.12, 95% CI 0.08 to 0.16, *p*<0.05) and Wuqinxi (SMD= -0.02, 95% CI -0.09 to -0.05, *p*<0.05), and the difference was statistically significant. Moreover, the Baduanjin+drug group (SMD= -0.14, 95%CI -0.23 to -0.05, *p*<0.01) was superior to the Baduanjin and the Taiji+drug group (SMD= -0.10, 95%CI -0.18 to -0.01, *p*<0.05), which was superior to Taiji (**Table 7**).

**Table 7:** Results of the network meta-analysis for femoral neck BMD.

	Drug group	Exercise training	Yijinjin	Usual care	Wuqinxi	Baduanjin	Taiji	Wuqinxi+ drug group	Taiji+drug group
Drug group									
Exercise training	-0.16 (-0.27,-0.06)								
Yijinjin	0.25 (0.14,0.37)*	-0.25 (-0.37,-0.12)*							
Usual care	-0.14 (-0.21,0.08)	0.03 (-0.03,0.08)	0.07 (0.01,0.19)*						
Wuqinxi	0.07 (-0.00,0.15)	-0.25 (-0.36,-0.13)*	0 (-0.09,0.09)	-0.04 (-0.07,-0.01)*					
Baduanjin	-0.03 (-0.10,0.03)	-0.23 (-0.31,-0.14)*	0.02 (-0.07,0.11)	-0.18 (-0.32,-0.19)*	-0.02 (-0.09,-0.01)*				
Taiji	0 (-0.07,0.06)	-0.10 (-0.17,0.05)	-0.07 (-0.12,0.05)*	-0.11 (-0.23,-0.07)*	-0.07 (-0.13,-0.04)*	0.12 (0.08,0.16)*			
Wuqinxi+ drug group	-0.02 (-0.07,0.02)	-0.10 (-0.19,0.02)	0.16 (0.03,0.29)*	-0.12 (-0.21,-0.02)*	-0.09 (-0.14,-0.05)*	0.11 (0.05,0.13)*	-0.08 (-0.17,0.02)		
Taiji+ drug group	-0.07 (-0.12,-0.02)*	-0.11 (-0.20,-0.01)*	0.14 (0.02,0.26)*	-0.14 (-0.22,-0.06)*	0.04 (-0.13,0.25)	0.12 (0.04,0.26)*	-0.1 (-0.18,-0.01)*	0.02 (-0.07,0.09)	
Baduanjin+ drug group	-0.08 (-0.16,-0.01)*	-0.15 (-0.25,-0.05)*	-0.02 (-0.23,0.11)	-0.27 (-0.32,-0.19)*	-0.09 (-0.21,-0.03)*	0.07 (-0.01,0.16)	-0.14 (-0.23,-0.05)*	-0.07 (-0.14,0.01)	-0.04 (-0.11,0.02)

shows that the data differ. *p< 0.05.

**Figure 6:**
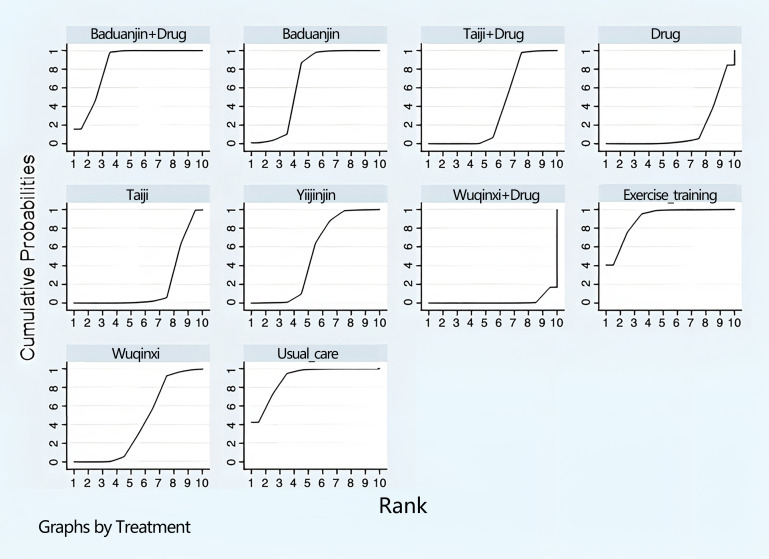
Ranking diagram of each intervention (femoral neck BMD).

The final ranking using SUCRA was as follows (**Figure 6**): Baduanjin+drug (90.2%) > Baduanjin (89.9%) > Taiji+drug (84.5%) > drug (66.7%) > Taiji (51.3%) > Yijinjin (42.4%) > Wuqinxi+drug (39.4%) > exercise training (19%) > Wuqinxi (14.8%) > usual care (1.9%).

#### Effect of TCE on Ward’s triangle BMD in postmenopausal women

**Figure 7:**
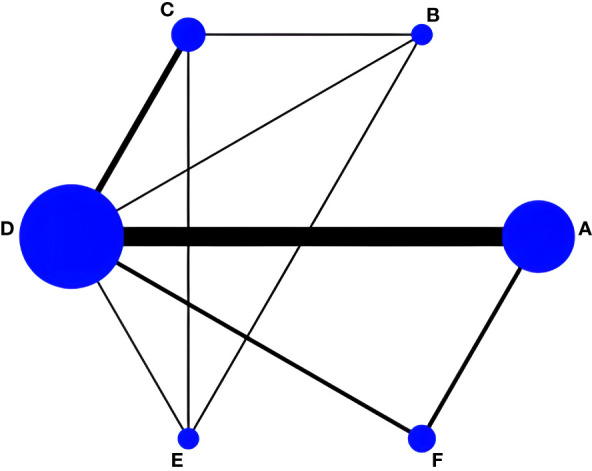
Network evidence for Ward’s triangle BMD. **(A)** Drug group; **(B)** Taiji+Drug group; **(C)** Taiji; **(D)** Baduanjin; **(E)** Wuqinxi; **(F)** Usual care.

LI tests and a net meta-analysis of the included data showed IF values ranging from 0.01 to 0.10 and a lower 95% CI of 0, indicating no significant inconsistency across the closed loop analyses using the consistency model. Thirteen RCT studies reported changes in Ward’s triangle bone density before and after the intervention, amounting to 1324 subjects; one included drug therapy as the control group, and 12 had usual care as the control group, with a mesh relationship between the included studies (**Figure 7**).

**Figure 8:**
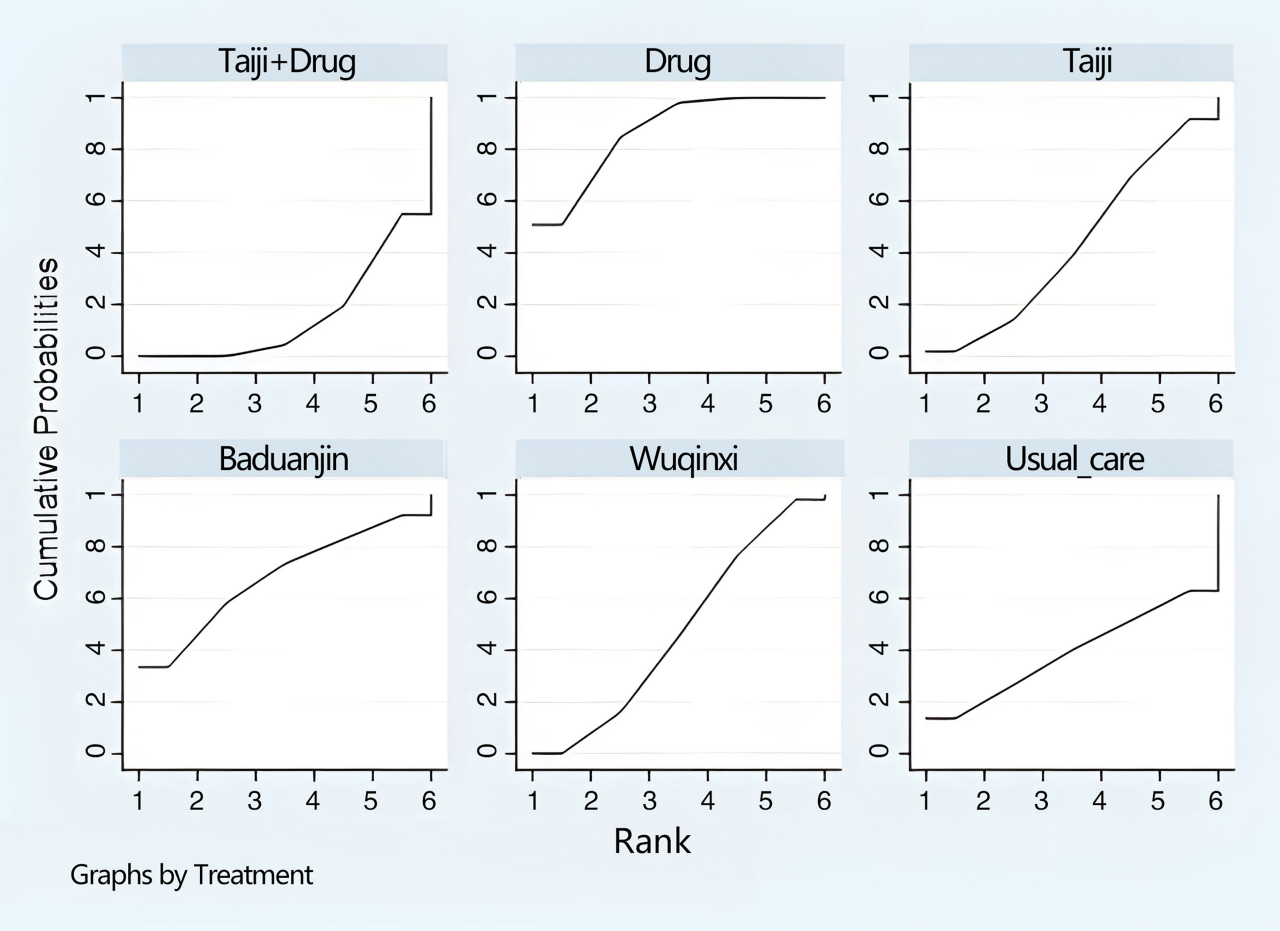
Ranking diagram of each intervention (Ward’s triangle BMD).

Of the six pairwise comparisons produced in the reticulated network analysis (**Table 8**), they were not statistically significantly different from the Taiji+drug group (SMD= -0.01,95%CI -0.06 to 0.04, *p >*0.05), Taiji (SMD= 0.02, 95%CI -0.01 to 0.05, *p*>0.05), and Wuqinxi (SMD= 0.03, 95%CI -0.04 to 0.09, *p*>0.05), but the drug group was superior to Baduanjin (SMD= 0.03, 95%CI 0.02 to 0.05, *p*<0.05). In addition, Taiji was superior to usual care (SMD= -0.06, 95%CI -0.13 to -0.03, *p*<0.05). In an indirect comparison, only Taiji was superior to Baduanjin (SMD= -0.02, 95% CI -0.04 to -0.01, *p*<0.05). The final SUCRA ranking was as follows (**Figure 8**): Taiji+drug (86%) > drug (68.7%) > Taiji (46.8%) > Baduanjin (41.2%) > Wuqinxi (39.8%) > usual care (15.5%).

**Table 8:** Results of the network meta-analysis for Ward’s triangle BMD.

	Drug group	Usual care	Wuqinxi	Baduanjin	Taiji
Drug group					
Usual care	0.02 (0.01,0.03)*				
Wuqinxi	0.03 (-0.04,0.09)	-0.01 (-0.07,0.01)			
Baduanjin	0.03 (0.02,0.05) *	-0.01 (-0.03,0.01)	0.01 (-0.06,0.07)		
Taiji	0.02 (-0.01,0.05)	-0.06 (-0.13,-0.03) *	0 (-0.07,0.06)	-0.02 (-0.04,-0.01) *	
Taiji+ drug group	0.01 (-0.04,0.06)	-0.07 (-0.10,-0.04) *	-0.02 (-0.09,0.05)	-0.03 (-0.08,0.02)	-0.02 (-0.06,0.03)

shows that the data differ. *p< 0.05.

## Discussion

To our knowledge, this article is the first to compare the effects of TCE (including drug treatment, usual care, exercise training, Taiji, Baduanjin, Wuqinxi, Yijinjin, and their combinations) on BMD in postmenopausal women by network analysis, using direct and indirect evidence to compare the effect of L-BMD, femoral neck BMD, and Ward’s triangle BMD in 33 randomized controlled trials (3658 postmenopausal women). The results showed that, compared with drug treatment, routine nursing, and exercise training, Taiji, Baduanjin, and Yijinjin can effectively prevent bone loss, and the effect of Wuqinxi needs to be further explored. Further, we distinguished between TCE and drug interventions to prevent the combined intervention from potentially exaggerating the impact of the intervention and causing bias in the experimental design.

In this study, all the traditional Chinese fitness exercise interventions improved the L-BMD status of postmenopausal women. Baduanjin+drug, Taiji+drug, Taiji, drug, and Yijinjin had better intervention effects. From the point of view of Chinese traditional fitness exercise alone (without combining their use with drugs), Taiji and Yijinjin achieved a more significant improvement in L-BMD and femoral neck BMD.

Many of the movements in Taiji required the subjects to adopt a semi-squatting posture, such as the Wild Horse Splitting Mane, Knee-Wrapping Reversing Stance, and Left and Right Ranging Sparrow’s Tail, etc. During the practice process, the subjects are required to adjust the stability of their body postures continuously. Moreover, when practicing, the subjects are required to sink their qi into the dantian and adopt abdominal breathing, which plays a role in exercising the core muscles of the torso, and long-term practice produces stress changes and increases bone mass of the lumbar spine (63), which in turn has a positive effect on L-BMD and femoral neck BMD. In Ward’s triangle BMD, Taiji+drug, drug, and Taiji were the optimal approaches for improving BMD symptoms in postmenopausal women, and this intervention was better than the control group, suggesting that Taiji has some muscle-strengthening effects. This finding was similar to previous studies by Zou, which showed that Taiji could improve L- BMD, femoral neck BMD, and Ward’s triangle BMD (18).

The effect of Yijinjing intervention was consistent with previous studies that concluded that Yijinjing was the best method for improving lumbar spine and femoral bone density via TCE (64). A previous study also showed that Yijinjing may be more beneficial for bone formation to improve BMD (65). A recent meta-analysis on osteoporosis in older people showed that, for femoral neck BMD and L-BMD, Yijinjing was better than Baduanjin and Wuqinxi, which yielded results of 0.02-0.05 g/cm^2^ and 0.07-0.08 g/cm^2^ (15). This was because the practice of Yijinjing uses the torso to drive the limbs to complete stretching, spreading, retracting, and rotating movements, which leads to the bones, muscles, ligaments, and joints moving from multiple angles, increasing the stimulation of the bones and improving their metabolic capacity (64). Through static stretching of the muscles and synergistic movement of the joints, we can stretch the tendons and bones, improve microcirculation and muscle mobility, and increase the body’s potential for change, thus strengthening the spleen, kidneys, and blood, as well as the tendons and bones. Furthermore, studies have shown that the estradiol level in older women increases significantly after Yijinjing, which positively affects bone density (66). However, considering the small number of studies focused on Yijinjing, its effectiveness in improving BMD in postmenopausal women has yet to be confirmed. Therefore, future high-quality original studies or systematic evaluations of the above interventions are needed.

In this study we identified a novel phenomenon: the effect of Baduanjin combined with medication on postmenopausal women’s L-BMD and femoral neck BMD was highly effective. Previous meta-analyses did not identify this, and we believe that this is because postmenopausal women were considered in this study. Related research confirms our other view that long-term regular Baduanjin exercises combined with medication can alleviate the lower back pain caused by osteoporosis in postmenopausal women and improve bone density. This is because the loss of bone density in postmenopausal women occurs mainly due to a decrease in the level of hormones synthesized by the ovaries, which disrupts the equilibrium of bone metabolism in the patient’s body, with more bone resorption than bone formation, thus accelerating the rate of bone loss in the patient. Pharmacological treatment supplements the hormones or may utilize osteoclast-inhibiting drugs (e.g., calcitonin, vitamin D, aluminophosphates, and calcium supplements) (14, 67). For this reason, postmenopausal women who choose Baduanjin+drug as an intervention should follow it long-term to treat L-BMD and femoral neck BMD. However, Taiji is relatively backward in overall alignment in L-BMD and femoral neck BMD. Some studies have pointed out that Taiji’s foot movements are variable in direction, slower, and lighter, similar to “stirring the feet” and “pointing the ground with a false toe step”, and that the ground reaction force on the soles of the feet is low during Taijiquan practice (68). However, previous studies have pointed to a positive correlation between the load applied to the bone and bone density, with the maximum force significantly increasing bone density. Therefore, compared to the Baduanjin interventions, Taiji is slightly less effective in decreasing BMD loss in postmenopausal women.

However, it is worth noting that this study found no improvement effect of Baduanjin on L-BMD in direct and indirect comparisons. As a traditional Chinese medicine fitness qigong, Baduanjin has slow and gentle movements, mainly used to regulate qi and blood, dredge meridians and channels, and then restrict the body’s internal organs and meridians. The movement of the joints needs to combine qi regulation and static force to play a role in strengthening the bones (69). Our findings may have been related to the differences in the trial subjects, the duration of the trial intervention, and the frequency of practice of Baduanjin. From the three articles included, it was found that the length and frequency of the intervention were short, which may have contributed to the poor results for the Baduanjin intervention in L-BMD in postmenopausal women. In contrast to the L-BMD results, the Baduanjin intervention alone significantly affected femoral neck BMD. The reasons for this phenomenon may be that the intervention used was a modified version of Baduanjin, the eighth stance of which in traditional Baduanjin is known as the toe posture, which can stimulate the immune system and enhance osteogenesis through the vertical pressure generated by the body’s gravity. However, the modified eighth stance of Baduanjin adds two training movements, such as tiptoeing and clapping. When the heel is suddenly put down, combined with the palm clapping action, the ankle, knee, and femur joints will increase the body’s gravity, hitting the ground and creating a reaction force, which will lead to vibration. Secondly, fewer studies were included, and it is possible that further research will confirm its effectiveness in improving femoral neck BMD in postmenopausal women. Therefore, future high-quality original studies or systematic evaluations of the above interventions based are needed.

Wuqinxi had a particular mitigating effect on BMD in postmenopausal women, and this finding was similar to the findings in previous studies (38, 70). Some studies have concluded that in elderly patients, Wuqinxi significantly improves BMD and prevents POP fractures compared with control groups, as it can enhance the bone formation index, reduce the rate of bone resorption, and achieve a dynamic and positive balance between osteogenesis and osteoblast genesis (35).

Some scholars have also shown that when older women practiced Wuqinxi for 24 weeks, lumbar spine L2-4, greater trochanter, Ward’s triangle, and femoral neck BMD improved to varying degrees. While not statistically significant, the static and dynamic balance in the left and right directions were improved, and the risk of falling was reduced (33). The reasons for these findings may be related to the duration, frequency, and intensity of Wuqinxi practice and the small sample size. A previous study showed that the protective BMD effect was difficult to observe in a 12-week intervention trial, as the bone remodeling cycle usually takes at least 24 weeks (71). However, there has yet to be a longitudinal study to demonstrate the effect of long-term exercise on changes in bone mineral density in older adults over time. Therefore, the comparison between different durations suggests interesting future directions for exploring protective bone density loss, and further research is needed.

This study has several limitations: (1) although we used 11 electronic databases, they were limited to Chinese and English articles, which may have led to a specific language bias; (2) we did not include VAS and serum ALP, as the number of studies on VAS and serum ALP were insufficient for network analysis, which may have weakened the strength of the evidence; (3) most of the studies were assigned inappropriately and non-double-blinded to exaggerate the effects of the treatment; (4) most studies lacked follow-up information, resulting in incomplete data on the effectiveness and safety of treatment regimens; and (5) this study focused on TCE interventions and discussed mainly single exercise interventions, distinguishing between combined TCE and drug interventions to avoid exaggerating or reducing the degree of effect of these interventions, but it remains unclear by which mechanism the combination of TCE and different medications affects BMD. Given the limitations of this study, there is a need to objectify and standardize the study design in this field to support the development of more high-quality literature, such as large-scale, prospective, double-blind RCTs.

## Conclusions

The results of the reticulated network analysis showed that all five traditional gong methods (Wuqinxi, Taiji, Baduanjin, Yijinjin, and Liuzijue) were effective in addressing BMD in postmenopausal women. The probability ranking showed that Taiji and Yijinjing alone have significant advantages, while Baduanjin still needs more studies to testify that it has substantial benefits if combined with medication. Therefore, Taiji or Baduanjin combined with medication may be preferred to effectively prevent and treat osteoporosis in postmenopausal women in clinical practice at this stage. However, the specific disease should be considered, along with the patient’s actual situation, to choose the patient’s recommended fitness qigong rationally and discriminatively.

## Date availability statement

The original contributions presented in the study are included in the article/**Supplementary Material**. Further inquiries can be directed to the corresponding author.

## Author contributions

SL: Conceptualization, Data curation, Resources, Visualization, Writing – original draft. SW: Data curation, Formal analysis, Methodology, Software, Writing – review & editing. JQ: Data curation, Software, Supervision, Validation, Writing – review & editing. LW: Data curation, Funding acquisition, Supervision, Writing – review & editing.
