# Supplementary material for: Effect of traditional Chinese fitness exercises on bone mineral density in postmenopausal women: a network meta-analysis of randomized controlled trials
Source: Front Endocrinol (Lausanne). 2024 Feb 6;15:1323595. doi: 10.3389/fendo.2024.1323595 (PMC10882717; doi:10.3389/fendo.2024.1323595)
Supplement: Supplementary file 2 [file Table_2.docx]

**Supplementary Table 1. Search items used in China National Knowledge Infrastructure**

| No. | Search items |
| --- | --- |
| 1 | (AB='女性''+'绝经期') |
| 2 | (AB='骨密度'+'骨质减少'+'骨疏松'+'骨量'+'骨质流失'+'骨病') |
| 3 | (AB='健身气功'+'中华传统功法'+'太极'+'八段锦'+'五禽戏'+'六字诀'+'易筋经'+'体育锻炼'+'运动') |
| 4 | #1 AND #2 AND #3 |

**Supplementary Table 2. Search items used in Embase.**

| No. | Search items |
| --- | --- |
| 1 | ‘Women’/exp OR ‘menopause’/exp OR postmenopausal*:ab,ti OR female*:ab,ti |
| 2 | ‘Bone mineral density’/exp OR ‘bone mass’/exp OR ‘osteoporosis’/exp OR ‘metabolic bone disease’/exp OR osteoporo*:ab,ti OR ‘bone loss’:ab,ti OR ‘low bone densit*’:ab,ti OR osteopenia*:ab,ti |
| 3 | ‘traditional Chinese exercise’/exp OR ‘Health Qigong’/exp OR ‘Qigong’/exp OR ‘Chi Kung’/exp OR ‘Taiji*’:ab,ti OR ‘Baduanjin*’:ab,ti OR Wuqinxi*:ab,ti OR Yijinjing:ab,ti OR Liuzijue*:ab,ti OR ‘Tai Chi*’:ab,ti |
| 4 | #1 AND #2 AND #3 |

**Supplementary Table 3. Search items used in PubMed.**

| No. | Search items |
| --- | --- |
| 1 | Women[MeSH Terms] |
| 2 | female[MeSH Terms] |
| 3 | postmenopausal*[Title/Abstract] |
| 4 | menopause*[Title/Abstract] |
| 5 | #1 OR #2 OR #3 OR #4 |
| 6 | Osteoporosis[MeSH Terms] |
| 7 | osteoporo*[Title/Abstract] |
| 8 | Bone Diseases, Metabolic[MeSH Terms] |
| 9 | "bone loss"[Title/Abstract] |
| 10 | "low bone densit*"[Title/Abstract] |
| 11 | osteopenia*[Title/Abstract] |
| 12 | "bone mineral density*"[Title/Abstract] |
| 13 | "bone mass*"[Title/Abstract] |
| 14 | #6 OR #7 OR #8 OR #9 OR #10 OR #11 OR #12 OR #13 |
| 15 | "traditional Chinese exercise*"[Title/Abstract] |
| 16 | "Health Qigong*"[Title/Abstract] |
| 17 | Qigong[MeSH Terms] |
| 18 | Chi Kung[MeSH Terms] |
| 19 | ‘Taiji*[Title/Abstract] |
| 20 | Baduanjin[Title/Abstract] |
| 21 | Wuqinxi*[Title/Abstract] |
| 22 | "Yijinjinge*"[Title/Abstract] |
| 23 | "Liuzijue*"[Title/Abstract] |
| 24 | #15 OR #16 OR #17 OR #18 OR #19 OR #20 OR #21 OR #22 |
| 25 | #5 AND #14 AND #24 |
